# Supplementary material for: Gene Expression Profiling of Olfactory Neuroblastoma Helps Identify Prognostic Pathways and Define Potentially Therapeutic Targets
Source: Cancers (Basel). 2021 May 21;13(11):2527. doi: 10.3390/cancers13112527 (PMC8196700; doi:10.3390/cancers13112527)
Supplement: Supplementary file 1 [file cancers-13-02527-s001.zip › cancers-1188855-suppl-figures.pdf]

Supplementary Materials: Gene Expression Profiling of Olfactory Neuroblastoma Helps Identify Prognostic Pathways and Define Potentially Therapeutic Targets

Chiara Romani, Eliana Bignotti, Davide Mattavelli, Anna Bozzola, Luigi Lorini, Michele Tomasoni, Laura Ardighieri, Vittorio Rampinelli, Alberto Paderno, Simonetta Battocchio, Cristina Gurizzan, Paolo Castelnuovo, Mario Turri-Zanoni, Carla Facco, Fausto Sessa, Alberto Schreiber, Marco Ferrari, Antonella Ravaggi, Alberto Deganello, Piero Nicolai, Michela Buglione, Davide Tomasini, Roberto Maroldi, Cesare Piazza, Stefano Calza and Paolo Bossi

Snapshot of enrichment results (Hallmark gene sets)

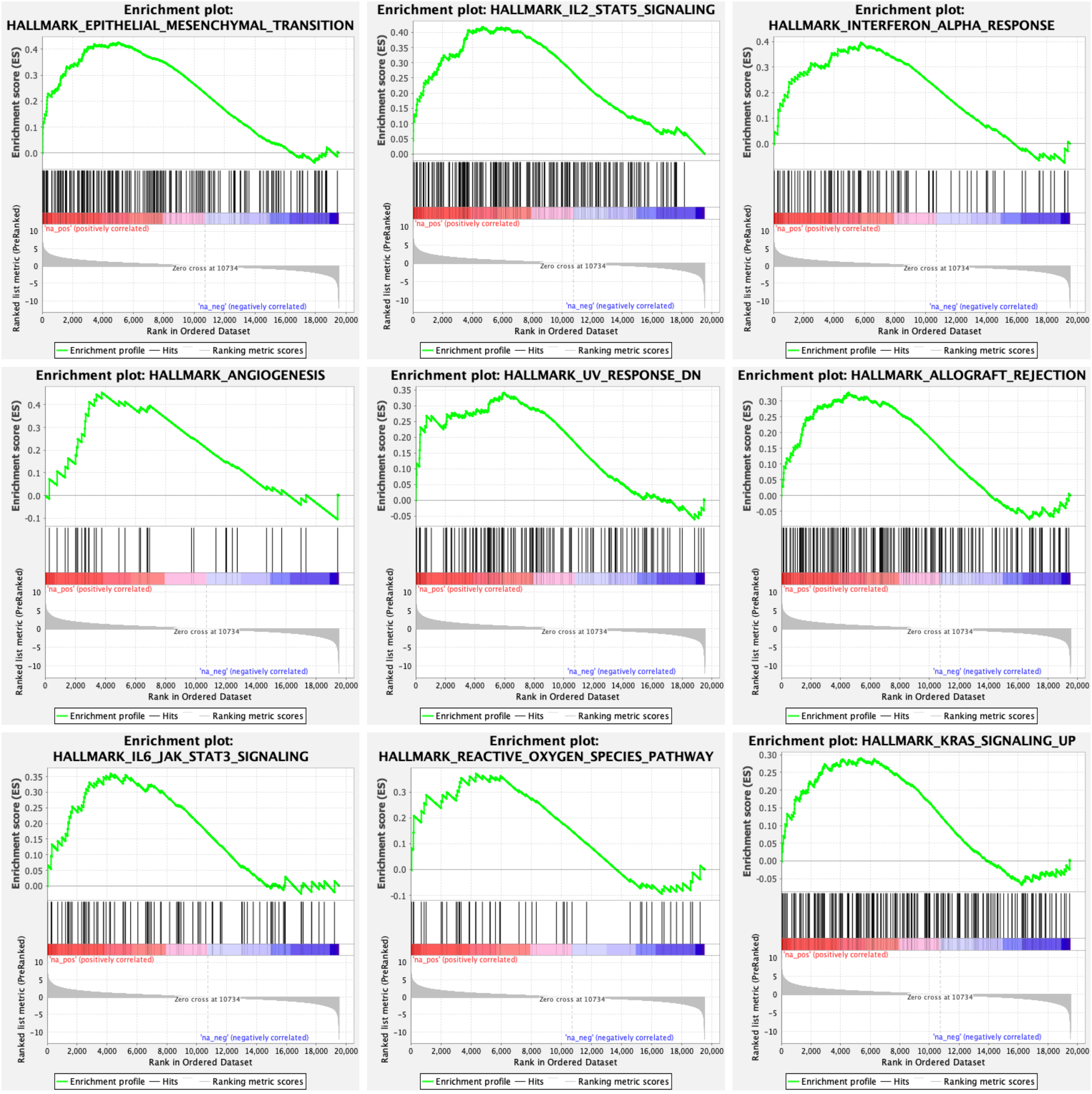

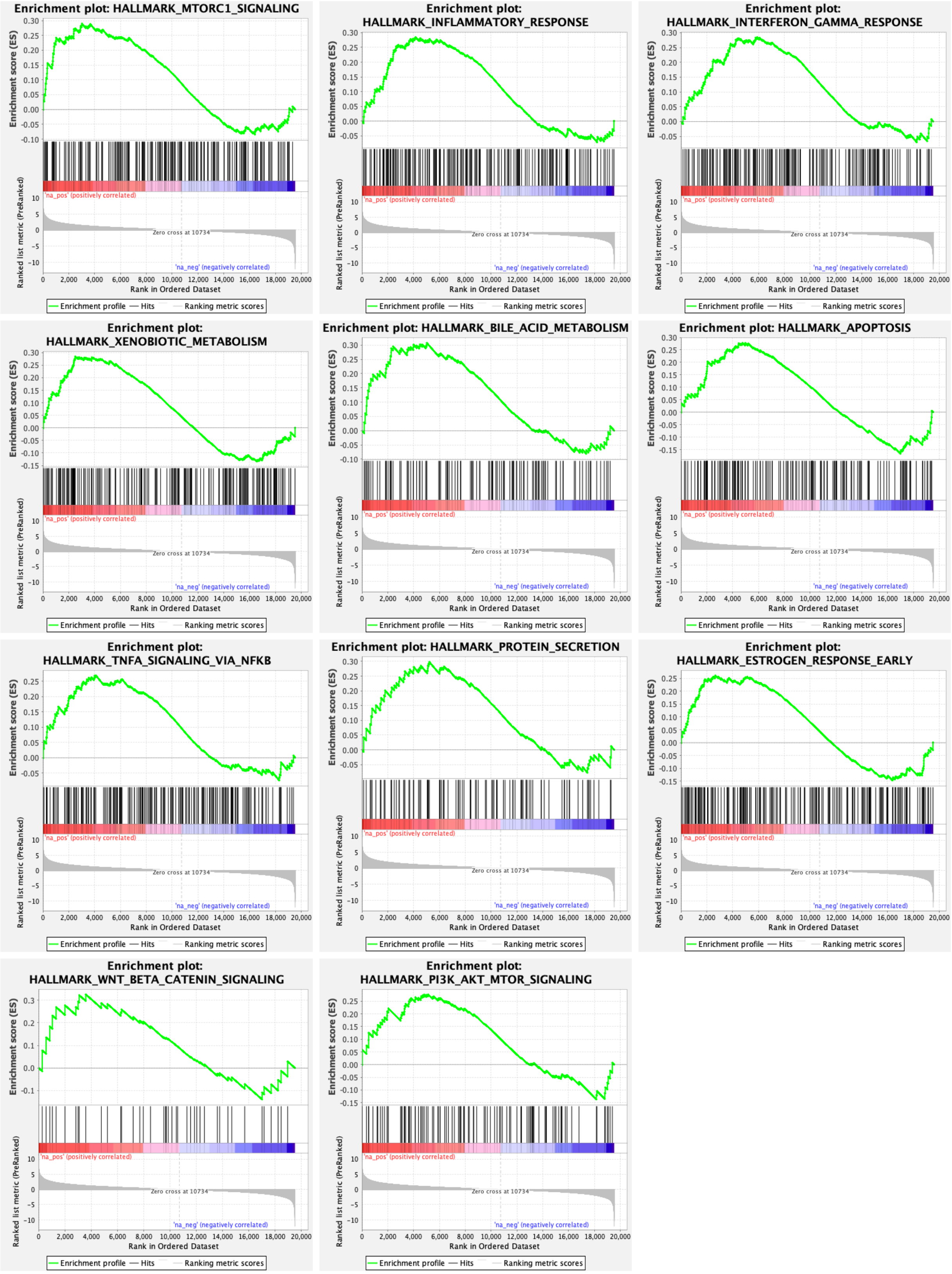

Figure S1. GSEA enrichment plots showing pathways associated with prognosis in ONB patients. The plots contain the profile of the running enrichment score (ES green line) and position of gene set members on the rank ordered list in GSEA (black strokes on the bottom).

Snapshot of enrichment results (GO gene sets)

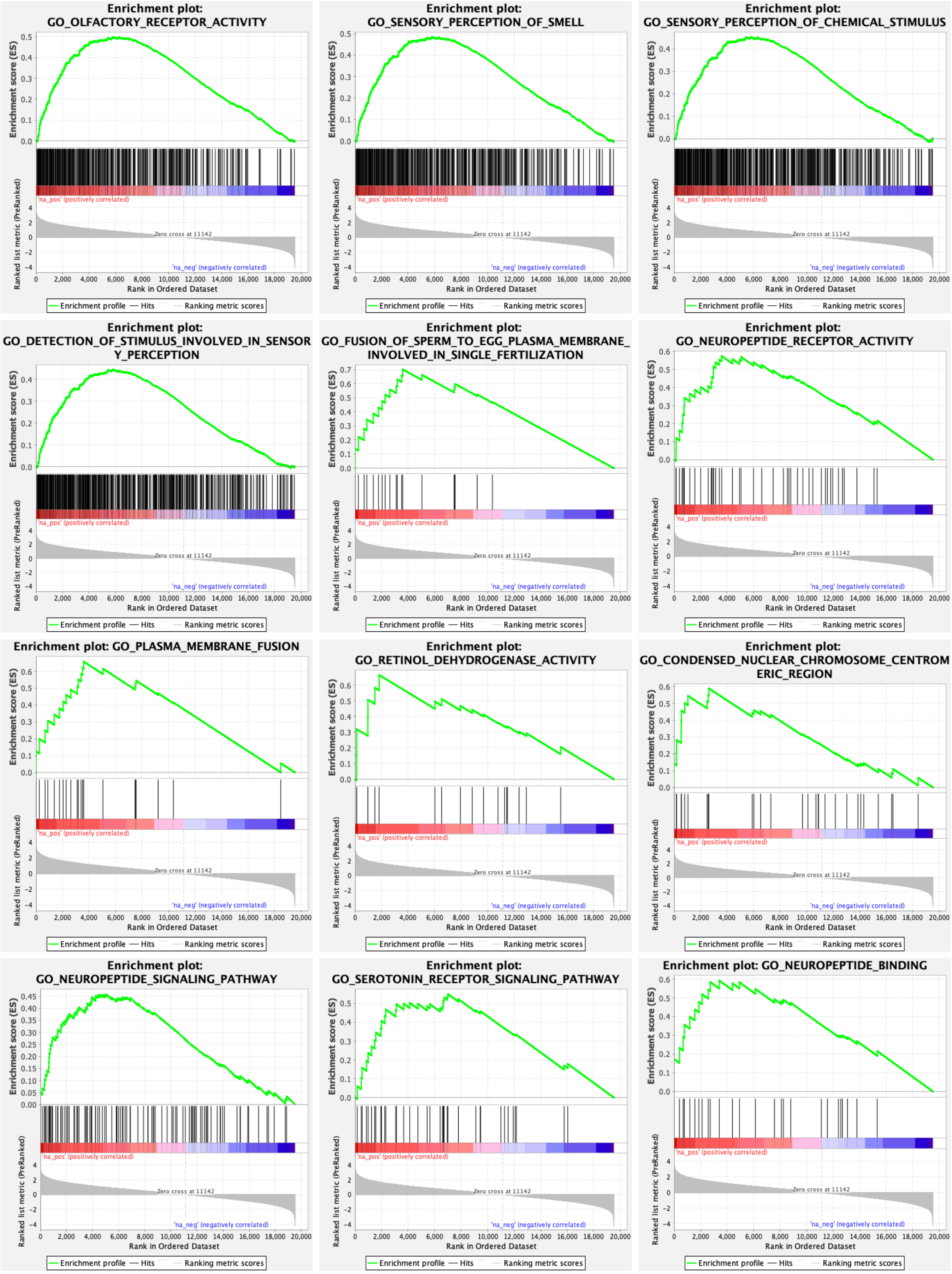

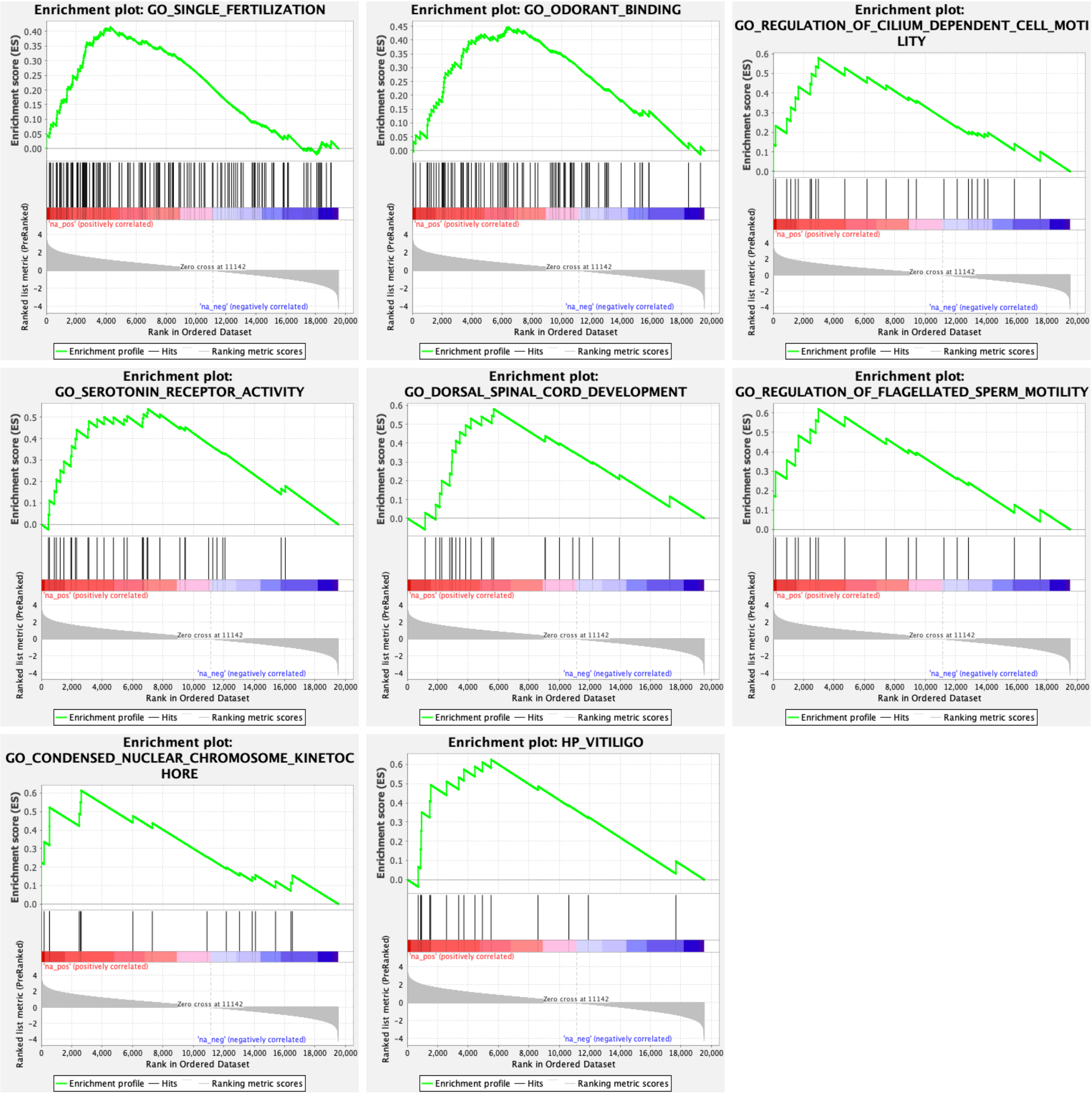

Snapshot of enrichment results (Hallmark gene sets)

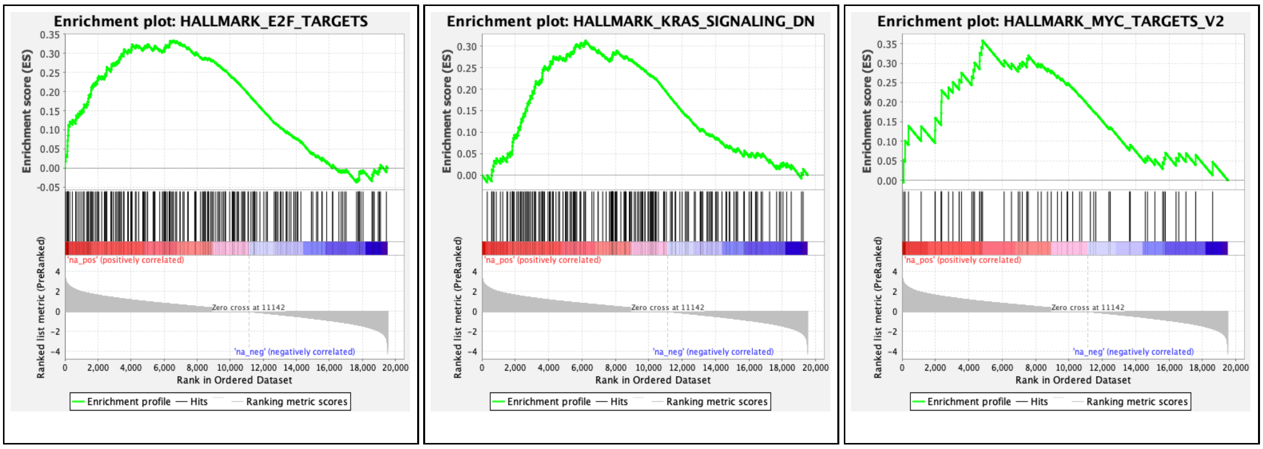

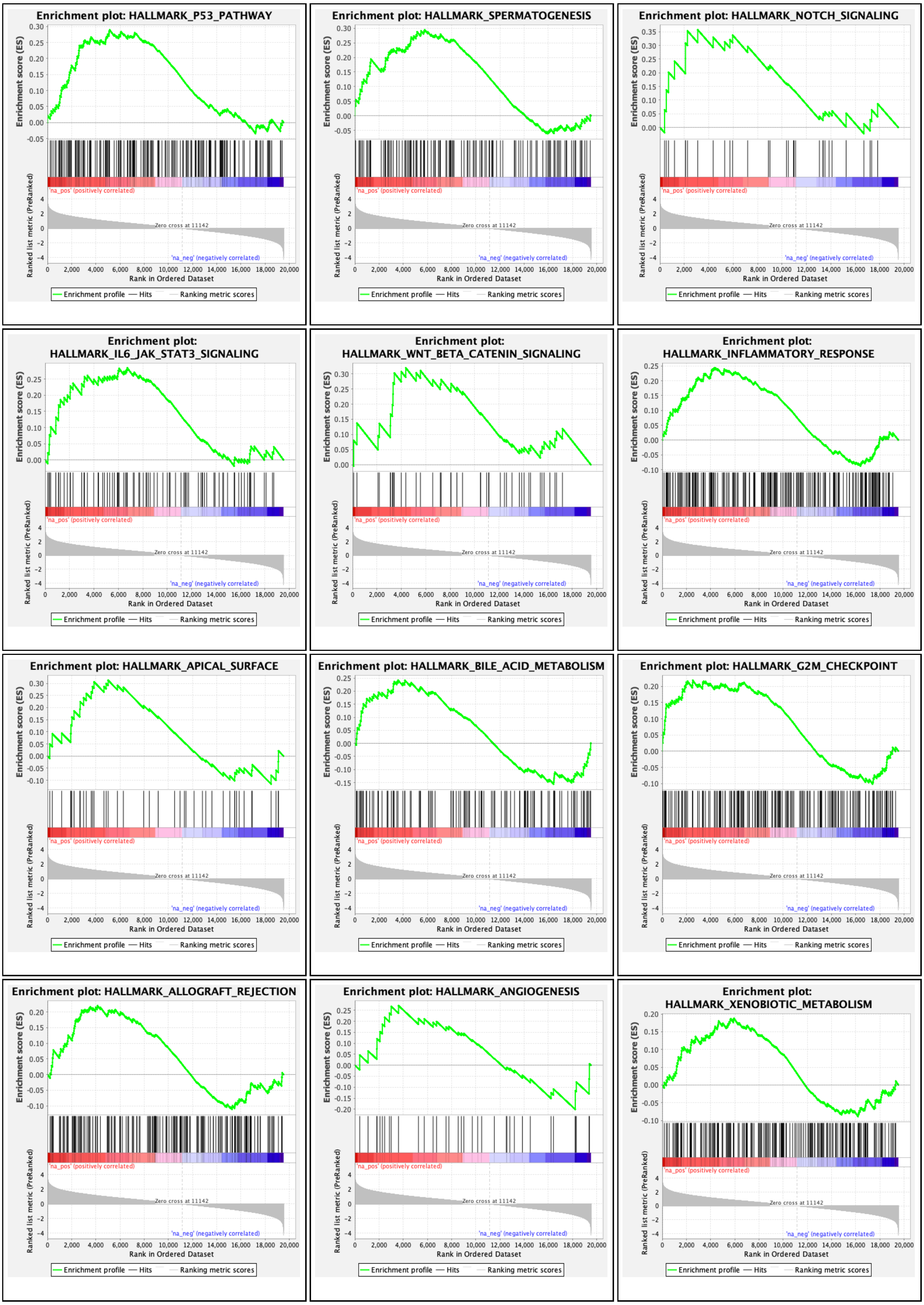
Enrichment plot: HALLMARK_P53_PATHWAY
Enrichment plot: HALLMARK_SPERMATOGENESIS
Enrichment plot: HALLMARK_NOTCH_SIGNALING
Enrichment plot: HALLMARK_IL6_JAK_STAT3_SIGNALING
Enrichment plot: HALLMARK_WNT_BETA_CATENIN_SIGNALING
Enrichment plot: HALLMARK_INFLAMMATORY_RESPONSE
Enrichment plot: HALLMARK_APICAL_SURFACE
Enrichment plot: HALLMARK_BILE_ACID_METABOLISM
Enrichment plot: HALLMARK_G2M_CHECKPOINT
Enrichment plot: HALLMARK_ALLOGRAFT_REJECTION
Enrichment plot: HALLMARK_ANGIOGENESIS
Enrichment plot: HALLMARK_XENOBIOTIC_METABOLISM
Enrichment score (ES)
Ranked list metric (PreRanked)
'na_pos' (positively correlated)
Zero cross at 11142
'na_neg' (negatively correlated)
Rank in Ordered Dataset
Enrichment profile
Hits
Ranking metric scores

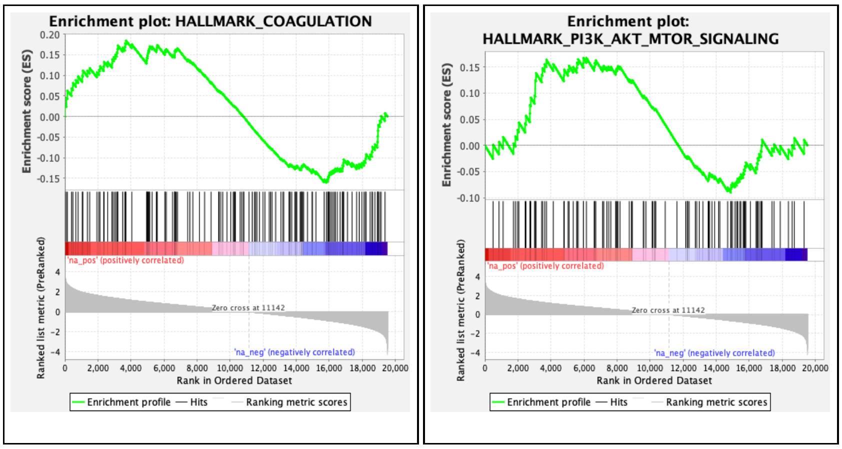

Positive enrichment in CK^{negative} patients group. The plots contain the profile of the running enrichment score (ES green line) and position of gene set members on the rank ordered list in GSEA (black strokes on the bottom).

Snapshot of enrichment results (GO gene sets)

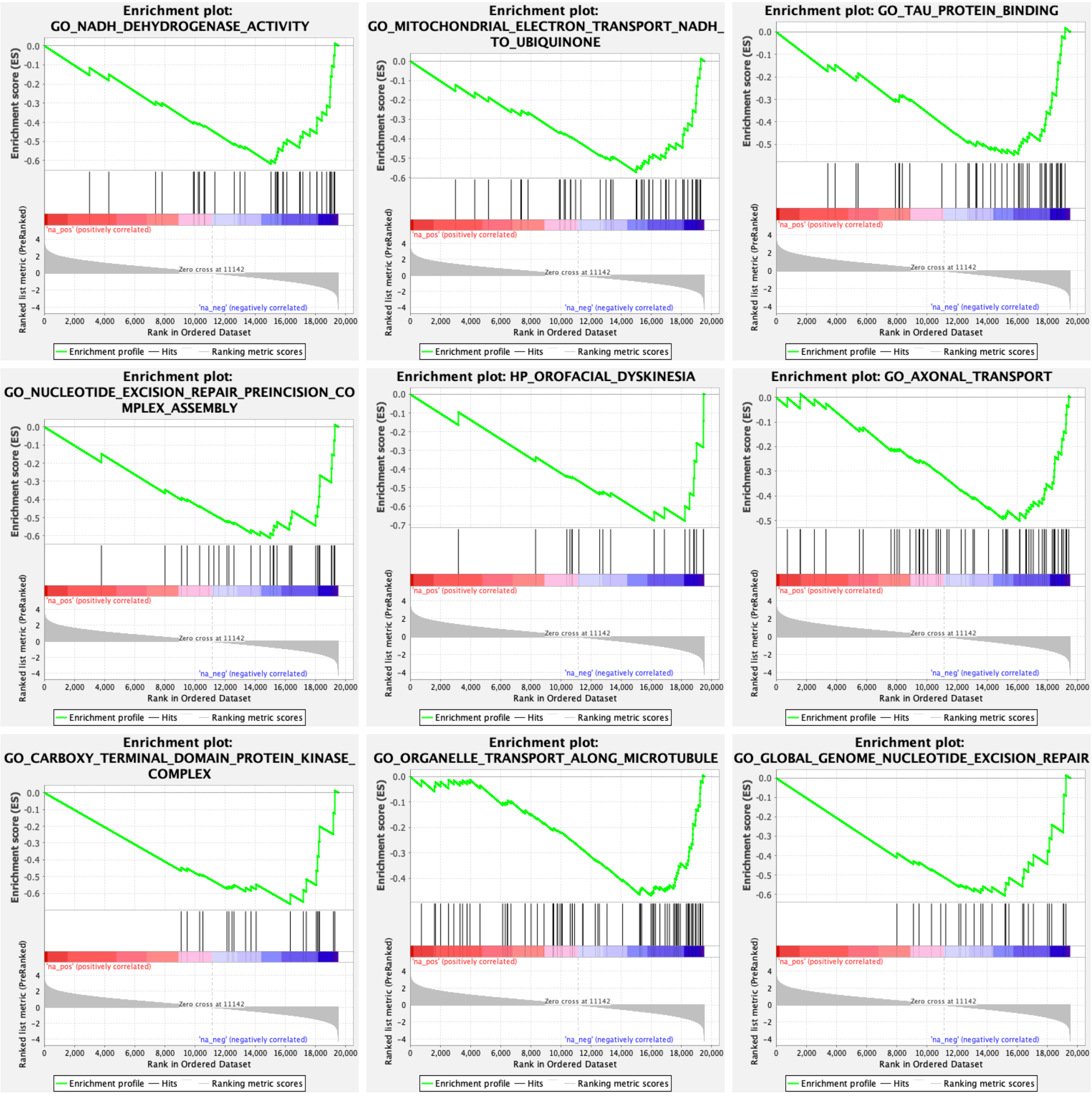

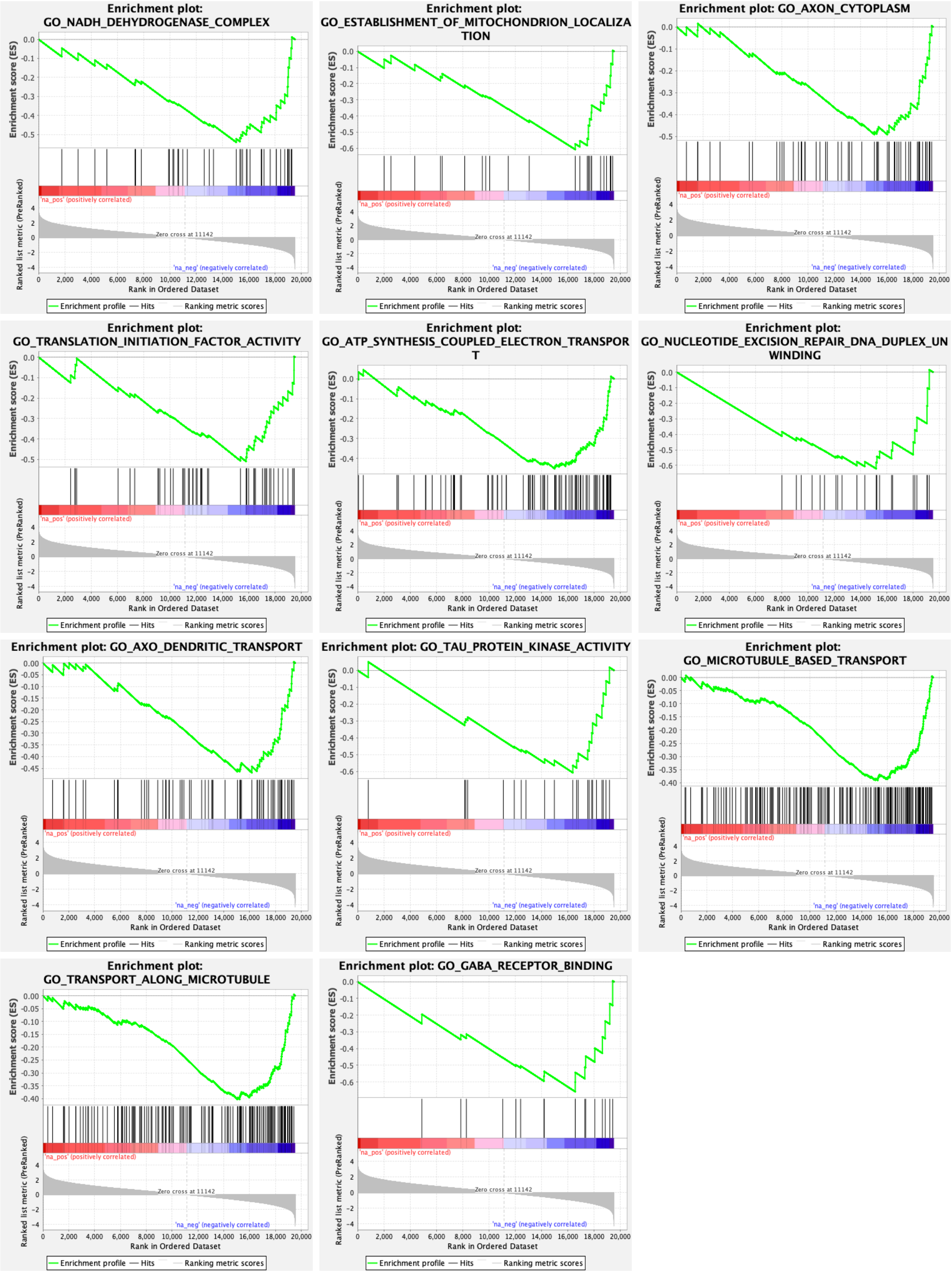

Snapshot of enrichment results (Hallmark gene sets)

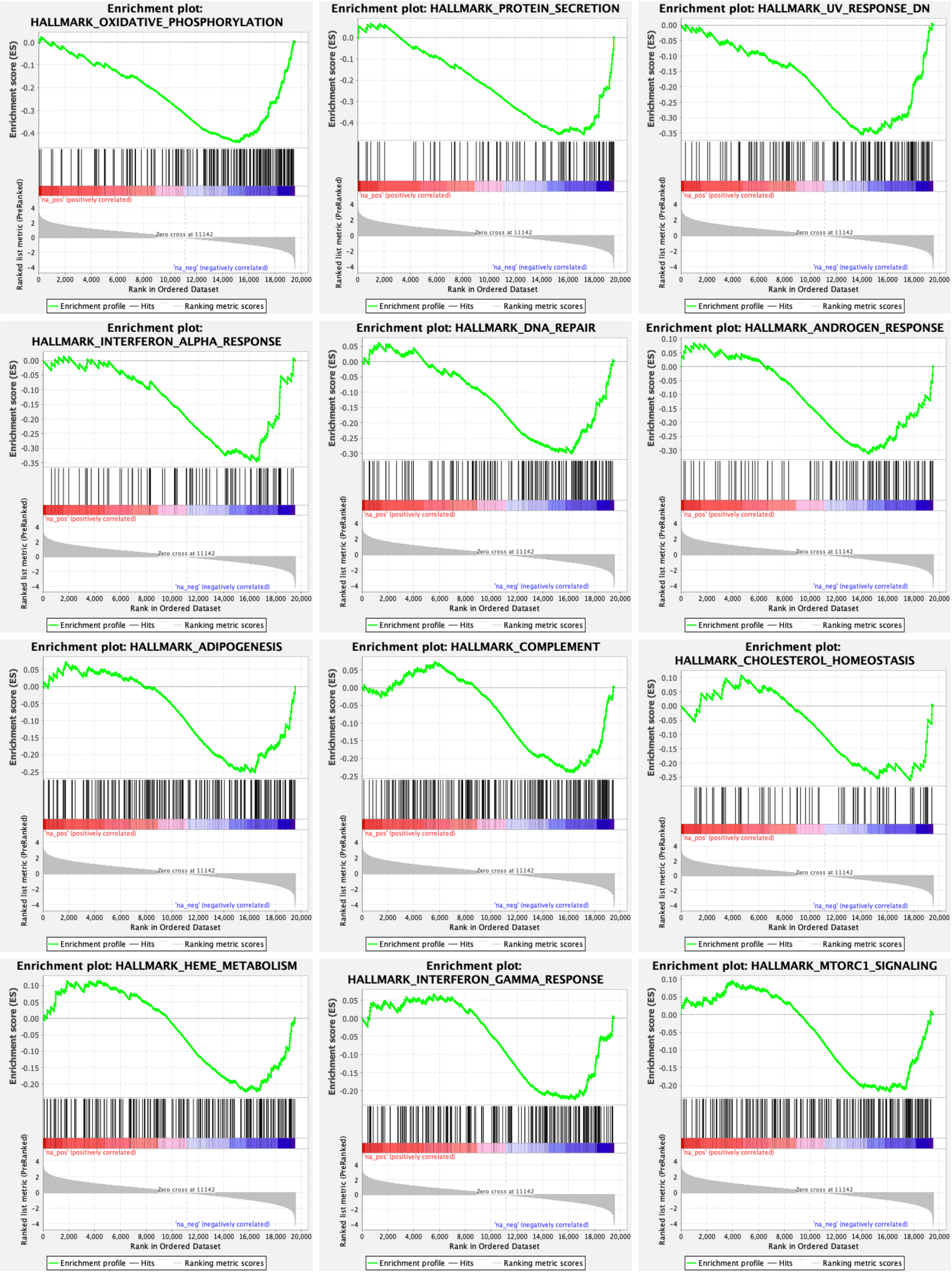
Enrichment plot: HALLMARK_OXIDATIVE_PHOSPHORYLATION
Enrichment plot: HALLMARK_PROTEIN_SECRETION
Enrichment plot: HALLMARK_UV_RESPONSE_DN
Enrichment plot: HALLMARK_INTERFERON_ALPHA_RESPONSE
Enrichment plot: HALLMARK_DNA_REPAIR
Enrichment plot: HALLMARK_ANDROGEN_RESPONSE
Enrichment plot: HALLMARK_ADIPOGENESIS
Enrichment plot: HALLMARK_COMPLEMENT
Enrichment plot: HALLMARK_CHOLESTEROL_HOMEOSTASIS
Enrichment plot: HALLMARK_HEME_METABOLISM
Enrichment plot: HALLMARK_INTERFERON_GAMMA_RESPONSE
Enrichment plot: HALLMARK_MTORC1_SIGNALING
Enrichment score (ES)
Ranked list metric (PreRanked)
'na_pos' (positively correlated)
Zero cross at 11142
'na_neg' (negatively correlated)
Rank in Ordered Dataset
Enrichment profile
Hits
Ranking metric scores

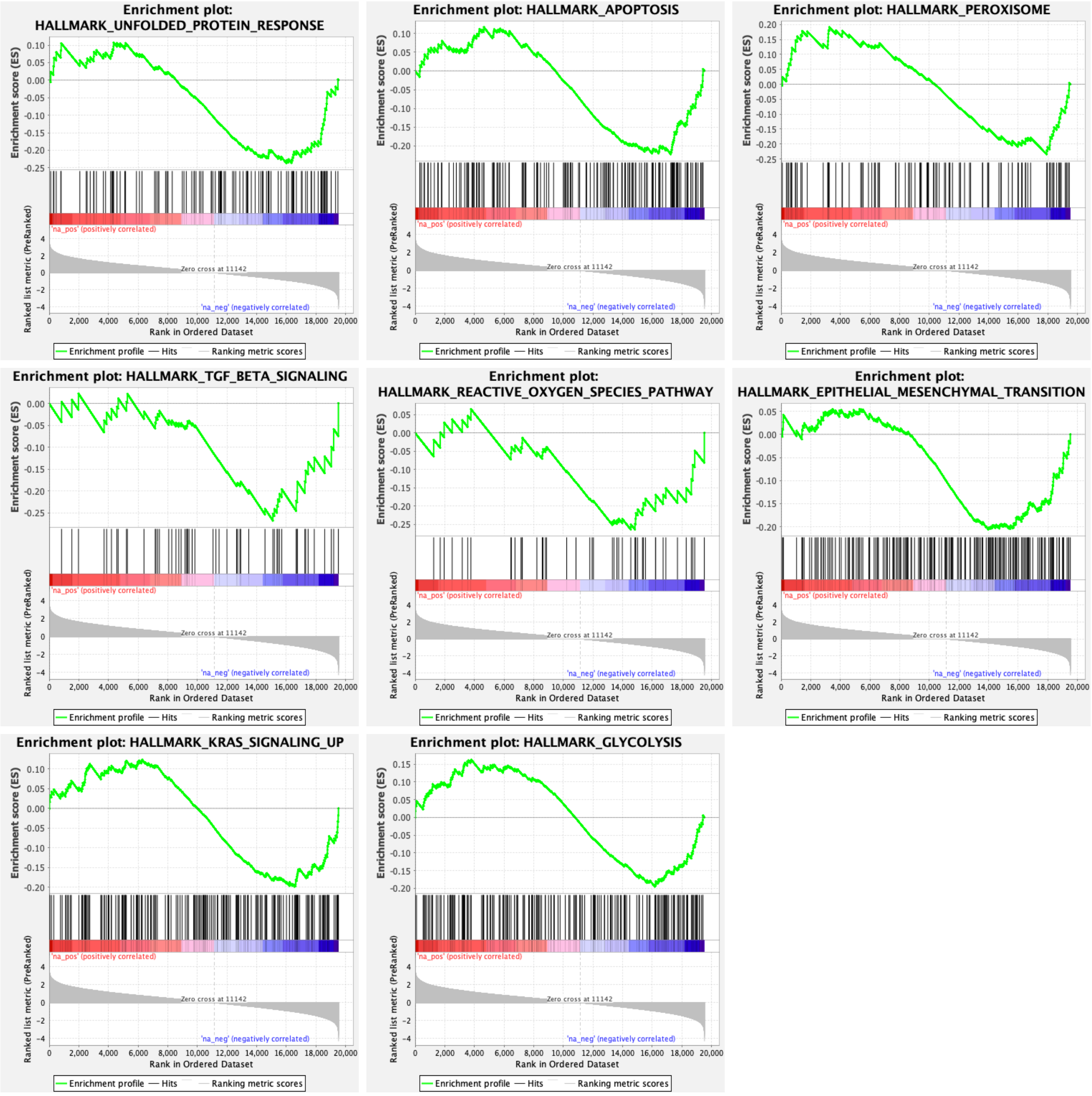

Figure S2. GSEA CK^{positive} vs CK^{negative} phenotype. Positive enrichment in CK^{positive} patients group. The plots contain the profile of the running enrichment score (ES green line) and position of gene set members on the rank ordered list in GSEA (black strokes on the bottom).
